# Cem Anos do Prêmio Nobel na Eletrocardiografia: Uma Tecnologia Ainda Essencial e Preparada para o Próximo Século

**DOI:** 10.36660/abc.20240494

**Published:** 2024-09-10

**Authors:** Eduardo M Vilela, Nuno Bettencourt

**Affiliations:** 1 Unidade Local de Saúde de Gaia e Espinho Vila Nova de Gaia Portugal Serviço de Cardiologia, Unidade Local de Saúde de Gaia e Espinho, Vila Nova de Gaia – Portugal; 2 Universidade do Porto Faculdade de Medicina Departamento de Medicina Porto Portugal Departamento de Medicina, Faculdade de Medicina, Universidade do Porto, Porto – Portugal; 3 Universidade do Porto Faculdade de Medicina Centro de Investigação Cardiovascular Porto Portugal Centro de Investigação Cardiovascular (UniC@RISE), Faculdade de Medicina, Universidade do Porto, Porto – Portugal

**Keywords:** Cardiologia, Eletrocardiografia, Síndrome Coronariana Aguda

## Willem Einthoven – breve revisão biográfica

**Figura 1:**
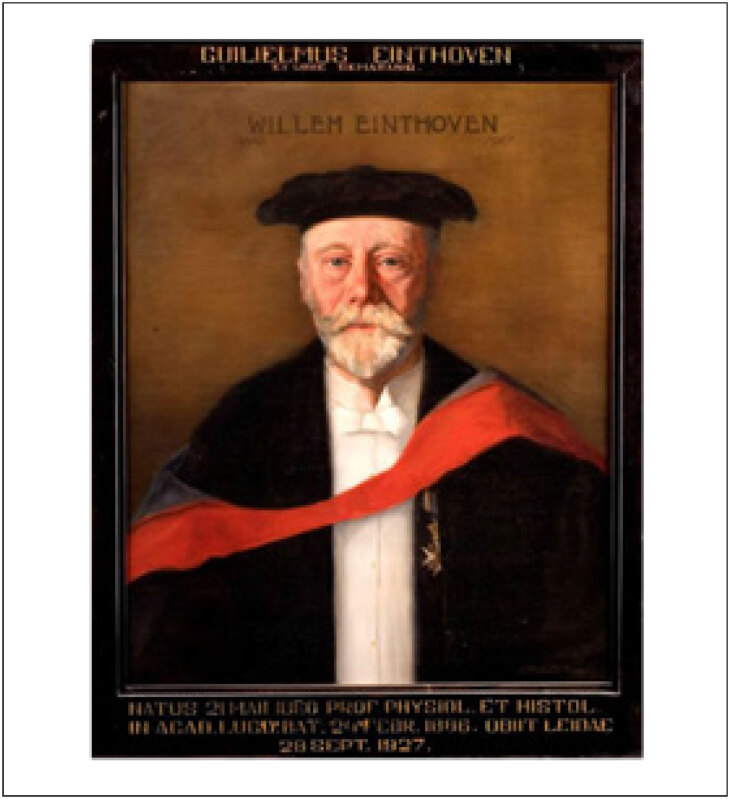
Retrato de Willem Einthoven. Reproduzido das Bibliotecas da Universidade de Leiden.^17^ Esta reprodução fotográfica é de domínio público.

Em 1924, o Prêmio Nobel de Fisiologia ou Medicina foi atribuído a Willem Einthoven pela "descoberta do mecanismo do eletrocardiograma" (ECG), evidenciando a relevância da investigação de Einthoven, que forneceu dados importantes sobre a fisiopatologia cardiovascular, e que viria a formar um dos pilares da Cardiologia moderna.^1^ Willem Einthoven nasceu em 1860 em Semarang, na ilha de Java, então parte das Índias Orientais Holandesas (na atual Indonésia), filho de um médico militar.^2 , 3^ Após a morte do pai, mudou-se com a família para os Países Baixos, onde deu continuidade a seus estudos, formando-se em medicina pela Universidade de Utrecht.^1 - 3^ Nesta fase de formação, ele foi profundamente influenciado pelo renomado fisiologista holandês Donders, que mais tarde o ajudou na obtenção de um cargo na Universidade de Leiden, onde permaneceu como professor de Fisiologia pelo resto de sua carreira ( Figura 1 ).^2 , 4^ Descrito como modesto, cortês e dedicado ao seu trabalho, além de suas pesquisas contínuas em ECG, Einthoven também era fluente em diversos idiomas e um defensor da importância da prática de exercícios físicos, tendo praticado remo e sendo conhecido por ir de bicicleta para o trabalho.^1 , 2 , 4 , 5^ Casou-se com uma de suas primas e teve quatro filhos, morrendo em 1927, aos 67 anos, por decorrência de um câncer.^3 - 5^ Apesar da árdua construção de uma base de pesquisa diversificada ao longo de décadas, incluindo desde os relatórios de Galvani e mais tarde de Matteucci, von Kolliker e Muller sobre correntes elétricas até o eletrômetro capilar de Lippman e a representação de Waller do "eletrograma" humano, a aplicação da tecnologia por Einthoven, juntamente com seus insights sobre sua possível utilidade futura, provou ser fundamental no aproveitamento de seu potencial.^1 - 5^

## O eletrocardiograma – desenvolvimento e primeiras aplicações

Em 1887, Augustus Desiré Waller (nascido em Paris em 1856, filho do cientista inglês Augustus Volney Waller, famoso por suas pesquisas sobre a degeneração nervosa, conhecida como degeneração Walleriana), trabalhando em Londres, foi creditado pela realização do primeiro ECG, chamado de "eletrograma" à época.^1 , 4 - 6^ Esse marco (curiosamente também realizado na época no buldogue de Waller, "Jimmy") abriu o caminho para um novo campo de aplicação, embora a técnica e a qualidade resultante do traçado limitassem sua aplicabilidade.^1 , 2 , 5 , 6^ Os refinamentos de Einthoven com a introdução do galvanômetro de corda, melhorando muito a sensibilidade de seus antecessores, permitiram uma representação detalhada da atividade elétrica do coração no início do século XX.^3 , 5 , 7^ Isto possibilitou a análise de diferentes formas de onda, como onda P, complexo QRS e onda T, representando diversos fenômenos fisiológicos (despolarização auricular, despolarização ventricular e repolarização, respetivamente), semelhante à avaliação realizada nos registos atuais.^5 , 7 , 8^ Embora fosse uma grande melhoria, o aparelho completo necessário para produzir um ECG precoce pesava cerca de 270 kg, demandando o uso de duas salas e cinco pessoas para sua operação.^1 , 4^ Além disso, um sistema de resfriamento de água era necessário para prevenir o superaquecimento.^1 , 5^ Demonstrando uma visão impressionante, Einthoven, seguindo a sugestão de Johannes Bosscha (também professor da Universidade de Leiden), posteriormente ligou o laboratório em que a máquina estava instalada a um hospital a cerca de 1,5 quilômetros de distância por meio de um cabo telefônico, produzindo assim o primeiro "telecardiograma", conforme publicado em um artigo seminal em 1906.^4 , 5 , 9 , 10^ Essa inovação mostra duas facetas que ressoam profundamente nos cenários contemporâneos: a relevância da telemedicina, mesmo no início daquilo que, posteriormente, evoluiria para o campo da medicina cardiovascular, e os benefícios de uma interação positiva entre o ambiente acadêmico e a prática clínica.^10^

Einthoven esteve, desde cedo, profundamente interessado nas possíveis aplicações clínicas do ECG, especialmente devido ao seu proeminente papel acadêmico.^1 - 3 , 6 , 9 , 10^ Diferentes padrões eletrocardiográficos foram rapidamente relatados, alguns associados a processos patológicos, como angina e arritmias.^4 , 9 - 11^ Embora esteja além do escopo deste artigo, é importante lembrar que muitos indivíduos contribuíram grandemente para o uso clínico do ECG.^11^ Entre eles, entretanto, Sir Thomas Lewis, da University College London teve um papel fundamental nesta fase, conforme reconhecido por Einthoven.^4 , 5 , 11^ Por outro lado, embora tenha apresentado uma primeira iteração desta técnica, Waller (pelo menos em um primeiro momento) não compartilhava do mesmo entusiasmo com as suas aplicações clínicas.^1 , 6 , 9^ Ainda assim, mais tarde também forneceria novos dados sobre a sua utilização, nomeadamente sob a forma de uma série de ECGs.^1 , 6^

## Da bancada ao leito – uso atual e perspectivas futuras para o eletrocardiograma

Ao longo da sua história ilustre, o ECG acompanhou diversos avanços importantes na medicina cardiovascular, incluindo modalidades de diagnóstico invasivas e não invasivas, bem como intervenções cada vez mais complexas.^4 , 7 , 12 - 14^ De forma notável, e considerando as melhorias tecnológicas desde o seu início – principalmente aquelas que compreendem a aquisição de sinais, a análise digital e o armazenamento, mas também o gigantesco salto em sua portabilidade quando comparado aos designs originais –, o ECG resistiu ao teste do tempo em uma infinidade de ambientes clínicos. Desde a sua utilização como exame de primeira linha, na avaliação de indivíduos com suspeita de síndromes coronarianas agudas, seu papel no estudo de arritmias e na seleção de indivíduos para intervenções como a terapia de ressincronização cardíaca, até sua combinação com outras metodologias (como o teste de esforço ou monitoramento contínuo), o ECG permanece onipresente na cardiologia contemporânea.^12 - 16^ Além disso, à medida que novas estruturas, como o advento e a rápida expansão da inteligência artificial, continuam transformando paradigmas anteriores, o uso do ECG como um modelo para novas avaliações enfatiza ainda mais sua relevância duradoura.^7^ Dada a visão de Einthoven do ECG como uma ferramenta clínica útil, é interessante formular hipóteses sobre qual seria sua reação ao testemunhar suas inúmeras aplicações nos dias de hoje. Além disso, ao recordarmos sua forte defesa do exercício físico, é interessante notar que a combinação destas duas facetas distintas, tão estimadas por Einthoven (o ECG e o exercício), tornou-se um dos testes mais extensivamente estudados e generalizados em toda a medicina cardiovascular: o teste ergométrico.^15 , 16^ Estes fatores constituem mais um elo entre os paradigmas atuais e uma das figuras mais eminentes da Cardiologia. Conforme aprimoramos nossa compreensão sobre a fisiologia cardiovascular, ao mesmo tempo em que desenvolvemos novas estratégias para otimizar o cuidado global ao paciente, é importante que haja uma reflexão sobre a sabedoria de nossos antepassados. Parafraseando a elegante expressão usada por Sir Isaac Newton, se conseguimos ver mais longe, é porque estamos sobre os ombros de gigantes.
