# Supplementary material for: Cem Anos do Prêmio Nobel na Eletrocardiografia: Uma Tecnologia Ainda Essencial e Preparada para o Próximo Século
Source: Arq Bras Cardiol. 2024 Sep 10;121(8):e20240494. [Article in Portuguese] doi: 10.36660/abc.20240494 (PMC12092061; doi:10.36660/abc.20240494)
Supplement: Translation

# Translation: One Hundred Years of the Nobel Prize for Electrocardiography: A Technology Still Essential and Fit for the Next Century

**DOI:** 10.36660/abc.20240494i

**Authors:** Eduardo M Vilela, Nuno Bettencourt

**Affiliations:** 1 Vila Nova de Gaia Portugal Serviço de Cardiologia, Unidade Local de Saúde de Gaia e Espinho, Vila Nova de Gaia – Portugal; 2 Porto Portugal Departamento de Medicina, Faculdade de Medicina, Universidade do Porto, Porto – Portugal; 3 Porto Portugal Centro de Investigação Cardiovascular (UniC@RISE), Faculdade de Medicina, Universidade do Porto, Porto – Portugal

**Keywords:** Cardiology, Electrocardiography, Acute Coronary Syndrome

## Willem Einthoven – A Brief Biographical Overview

In 1924, the Nobel prize for Physiology or Medicine was awarded to Willem Einthoven for "the discovery of the mechanism of the electrocardiogram" (ECG). This prestigious award illustrated the relevance of Einthoven's research, which provided crucial data on cardiovascular pathophysiology, and that would go on to form one of the pillars of modern Cardiology.^1^Willem Einthoven was born in 1860 in Semarang, on the island of Java, then part of the Dutch East Indies (now Indonesia), the son of a military physician.^2 , 3^After his father's death, he moved with his family to the Netherlands, where he pursued his studies, earning a medical degree at the University of Utrecht.^1 - 3^During this formative period, he was deeply influenced by the renowned Dutch physiologist Donders, who later supported him in obtaining a position at the University of Leiden, where he remained as professor of Physiology for the rest of his career ( Figure 1 ).^2 , 4^Described as modest, courteous, and devoted to his work, besides his continued research in the ECG, Einthoven was also fluent in several languages and a proponent of physical exercise, practicing rowing and being known for riding his bicycle to work.^1 , 2 , 4 , 5^He married one of his cousins and had four children, passing away in 1927 at the age of 67 from cancer.^3 - 5^Despite building on a diverse research background over decades, from Galvani's and later Matteucci's, von Kolliker and Muller's reports on electrical currents to Lippman's capillary electrometer and Waller's depiction of the human "electrogram," Einthoven's application of technology, coupled with far-reaching insights into its potential utility, proved pivotal in harnessing its potential.^1 - 5^

**Figure 1:** Portrait of Willem Einthoven. Reproduced from Leiden University Libraries.^17^This photographic reproduction falls within the public domain.

## The Electrocardiogram – Development and Early Applications

In 1887, Augustus Desiré Waller (born in Paris in 1856, the son of English scientist Augustus Volney Waller, renowned for his research on nerve degeneration eponymously celebrated as Wallerian degeneration), working in London, was credited with performing the first ECG, then referred to as the "electrogram".^1 , 4 - 6^This landmark (curiously also at the time performed on Waller's bulldog, "Jimmy") paved the way for a novel field, though the technique and the quality of the tracings limited its applicability.^1 , 2 , 5 , 6^Einthoven's refinements with the introduction of the string galvanometer, greatly improving the sensitivity of its predecessors, allowed for a detailed representation of the heart's electrical activity at the beginning of the twentieth century.^3 , 5 , 7^This advancement allowed for the analysis of different waveforms, such as the P wave, QRS complex, and T wave, representing several physiological phenomena (atrial depolarization, ventricular depolarization, and repolarization, respectively), similar to the assessment performed on modern-day recordings.^5 , 7 , 8^While a major improvement, the full apparatus necessary to produce an early ECG weighed around 270 kilograms, requiring two rooms and five individuals in its operation.^1 , 4^Additionally, a water cooling system was necessary to prevent overheating.^1 , 5^Showing remarkable vision, Einthoven, following the suggestion of Johannes Bosscha (also a professor at the University of Leiden), later connected the laboratory housing the machine to a hospital *circa* 1.5 kilometers away via a telephone cable, thus producing the first "telecardiogram," as documented in a seminal article in 1906).^4 , 5 , 9 , 10^This innovation shows two facets that resonate deeply with contemporary settings: the relevance of telemedicine, even at the dawn of what would evolve into the field of cardiovascular medicine, and the benefits of a positive interaction between academia and clinical practice.^10^

Notably, particularly given his prominent academic role, Einthoven was keenly interested in the possible clinical applications of the ECG from an early stage.^1 - 3 , 6 , 9 , 10^Different electrocardiographic patterns were soon being reported, some associated with pathological processes such as angina and arrhythmias.^4 , 9 - 11^Though beyond the scope of this article, it should be recalled that several individuals contributed greatly to the clinical use of the ECG.^11^Among these, however, Sir Thomas Lewis at the University College London had a key role at this stage, as acknowledged by Einthoven.^4 , 5 , 11^On the other hand, although having presented a first iteration of this technique, Waller (at least at first) did not share the same enthusiasm with its clinical applications.^1 , 6 , 9^Nonetheless, later on he would also provide new data on its use, namely in the form of a series of ECGs.^1 , 6^

## From Bench to Bedside – Current Use and Future Perspectives of the Electrocardiogram

Over the course of its illustrious history, the ECG has accompanied several major breakthroughs in cardiovascular medicine. These encompass both invasive and non-invasive diagnostic modalities, as well as increasingly complex interventions.^4 , 7 , 12 - 14^Remarkably, and while taking into consideration the technological improvements since its inception – namely those comprising signal acquisition, digital analysis, and storage, but also the giant leap in portability when compared to original designs –, the ECG has stood the test of time as a pivotal test across a plethora of clinical settings. From its use as a first-line test in assessing individuals with suspected acute coronary syndromes to its role in studying arrhythmias and selecting individuals for interventions such as cardiac resynchronization therapy, and its combination with other methodologies (such as in the case of exercise stress testing or continuous monitoring), the ECG remains ubiquitous in contemporary cardiology.^12 - 16^Moreover, as novel frameworks, such as the advent and rapid expansion of artificial intelligence, continue to disrupt prior paradigms, the use of the ECG as a blueprint for novel assessments further emphasizes its enduring relevance.^7^Given Einthoven's view of the ECG as a valuable clinical tool, it is interesting to hypothesize on what his reaction would be upon seeing its current manifold uses. In addition, recalling his strong advocacy for physical exercise, it is interesting to note that the combination of these two distinct facets so dear to Einthoven (the ECG and exercise) has rendered one of the most extensively studied and generalized tests across cardiovascular medicine: the exercise stress test.^15 , 16^These provide yet another link between current paradigms and one of cardiology's most eminent figures. As we deepen our understanding of cardiovascular physiology while developing novel strategies to optimize overall patient care, it is valuable to remember the wisdom of those who came before us. To paraphrase Sir Isaac Newton's elegant expression, if we can see further, it is by standing on the shoulders of giants.
